# Resting motor threshold in the course of hand motor recovery after stroke: a systematic review

**DOI:** 10.1186/s12984-021-00947-8

**Published:** 2021-11-03

**Authors:** Jitka Veldema, Dennis Alexander Nowak, Alireza Gharabaghi

**Affiliations:** 1 Institute for Neuromodulation and Neurotechnology, Department of Neurosurgery and Neurotechnology, University Hospital and University of Tübingen, Otfried-Mueller-Str.45, 72076 Tübingen, Germany; 2 Department of Neurology, VAMED Hospital Kipfenberg, Konrad-Regler-Straße 1, 85110 Kipfenberg, Germany

**Keywords:** Resting motor threshold, Hand motor recovery, Stroke

## Abstract

**Background:**

Resting motor threshold is an objective measure of cortical excitability. Numerous studies indicate that the success of motor recovery after stroke is significantly determined by the direction and extent of cortical excitability changes. A better understanding of this topic (particularly with regard to the level of motor impairment and the contribution of either cortical hemisphere) may contribute to the development of effective therapeutical strategies in this cohort.

**Objectives:**

This systematic review collects and analyses the available evidence on resting motor threshold and hand motor recovery in stroke patients.

**Methods:**

PubMed was searched from its inception through to 31/10/2020 on studies investigating resting motor threshold of the affected and/or the non-affected hemisphere and motor function of the affected hand in stroke cohorts.

**Results:**

Overall, 92 appropriate studies (including 1978 stroke patients and 377 healthy controls) were identified. The analysis of the data indicates that severe hand impairment is associated with suppressed cortical excitability within both hemispheres and with great between-hemispheric imbalance of cortical excitability. Favorable motor recovery is associated with an increase of ipsilesional motor cortex excitability and reduction of between-hemispheric imbalance. The direction of change of contralesional motor cortex excitability depends on the amount of hand motor impairment. Severely disabled patients show an increase of contralesional motor cortex excitability during motor recovery. In contrast, recovery of moderate to mild hand motor impairment is associated with a decrease of contralesional motor cortex excitability.

**Conclusions:**

This data encourages a differential use of rehabilitation strategies to modulate cortical excitability. Facilitation of the ipsilesional hemisphere may support recovery in general, whereas facilitation and inhibition of the contralesional hemisphere may enhance recovery in severe and less severely impaired patients, respectively.

## Introduction

Stroke is the leading cause of long-term disability in adults world-wide [1]. In consequence, rehabilitation and optimized care of stroke survivors is of high socio-economic priority. Motor impairment is the most common clinical deficit after stroke [1] and its recovery usually remains incomplete. Six months after the cerebro-vascular incident 60 to 70 percent of stroke survivors suffer from motor impairment of one hand which significantly impacts disability and activities of daily living [2, 3]. Up to now, tens of studies have shown that motor recovery after stroke is accompanied by reorganization of the functional network architecture within both the lesioned and the non-lesioned hemisphere [4, 5]. Nevertheless, the mechanism underlying recovery of motor function after a focal lesion is still not sufficiently understood.

Transcranial magnetic stimulation (TMS) is a neurophysiological method often used to probe neural processing related to hand motor function/recovery after stroke. A comprehensive analysis of these data may help to foster our understanding of the neurophysiological changes in cortical excitability accompanying motor recovery and, at the same time, may contribute to optimize stroke rehabilitation. For this reason, we performed a review on the relationship of changes in corticospinal excitability within the ipsi- and contralesional hemisphere (as measured by TMS) and the functional outcome of the affected hand after stroke. This review summarizes current data on resting motor threshold and hand motor function over the course of recovery after stroke and compares these data with the data of healthy subjects. Following issues need to be clarified: (1) Is the cortical excitability of the ipsi- and the contralesional hemisphere in stroke patients higher or lower in comparison to a healthy brain? (2) Is the between-hemispheric balance of cortical excitability in stroke subjects shifted toward the contra- or ipsilesional hemisphere? (3) Is there a relationship between the level of cortical excitability within either hemisphere and the between-hemispheric imbalance? (4) Is there a relationship between the level of cortical excitability within either hemisphere and/or the between-hemispheric imbalances, and the motor function/motor recovery of the affected hand?

### Neural plasticity following stroke

A focal brain lesion causes disturbance of functional and structural architecture within both the ipsilesional and contralesional hemisphere [4, 5]. Motor recovery results from the reorganization of neural interconnection within intact neuron pools, and causes alterations of movement-related neural activity within perilesional and more distant brain areas [4, 5]. This process is thought to compensate and adjust functional brain capacities to the new situation. “Adaptive/positive plasticity” means reorganization within neural tissue to optimize neural resources for recovery of function. However, such brain plasticity is not always “adaptive/positive”. The idea of “maladaptive/negative plasticity”, which may hamper motor recovery after stroke, is based on the theory of interhemispheric rivalry [6, 7]. In a healthy brain, neural activity in the motor areas of both hemispheres is functionally coupled and equally balanced in terms of mutual inhibitory control. An active movement of a hand is associated with an enhanced neural activity in contralateral motor areas and increased inhibitory influence toward homologous areas of the ipsilateral hemisphere [8, 9]. In stroke patients, a shift of the between-hemispheric balance detrimental to the affected hemisphere can be observed. Several fMRI and PET studies have shown that during an active movement of the affected hand there is increased neural activity within motor areas of both the lesioned and the non-lesioned hemisphere and describe a link to hand motor disability [10–13]. Patients with a favorable functional outcome show lateralized activation within the contralateral hemisphere (comparably to healthy subjects) during active movement of the affected hand. In contrast, patients with a poor motor outcome show bilateral recruitment of motor-related brain regions when moving the affected hand [10–13]. Based on this data, a maladaptive role of the contralesional (i.e., ipsilateral) hemisphere for motor recovery after stroke has been postulated. It has been assumed that the “overactive” non-lesioned hemisphere exerts an increased inhibitory influence towards the homologous areas of the lesioned hemisphere and hampers in this way the motor recovery of the affected hand. However, the general validity of this theory is still under debate. In contrast to fMRI and PET trials [10–13], TMS studies showed no clear evidence for increased excitability of the unaffected hemisphere or imbalanced interhemispheric inhibition. Moreover, no differences were detected between the unaffected hemisphere and healthy brains [14]. Furthermore, recent EEG-TMS studies provide contrasting findings with regard to interhemispheric interactions in chronic stroke cohorts. One study detected increased TMS-evoked interhemispheric beta coherence during ipsilesional M1 stimulation. This was associated with reduced intracortical inhibition within both the ipsi-and the contralesional hemisphere as compared to healthy subjects [15]. In contrast, another study found decreased TMS-evoked interhemispheric beta coherence during ipsilesional M1 stimulation and detected a correlation to the amount of hand motor disability [16]. Both studies have not found any relevant differences between the contralesional hemisphere in stroke patients in comparison to healthy controls. These findings indicate that the changes of neural processing following stroke are complex and not well understood.

### Resting motor threshold

Over the past decades, tens of TMS-studies have investigated reorganization within the motor cortex after stroke as well as its relationship to hand motor recovery. We performed a comprehensive review on the resting motor threshold measure (an objective assessment of cortical excitability) and its relationship to motor rehabilitation. The resting motor threshold (rMT) is considered as the stimulus intensity that causes a “minimum motor response” in a resting muscle during single transcranial magnetic stimulation (TMS) pulses applied over the “motor hotspot” [17]. In literature, the “minimum motor response” is defined as the lowest stimulator output intensity that elicits a motor evoked potential (MEP) with a peak-to-peak amplitude of at least 50 µV in at least 50% of 8, 10 or 20 consecutive stimuli [17]. The “motor hotspot” is defined as the position on the scalp where the greatest amplitude and minimum latency of the motor evoked potential can be elicited [17]. A low resting motor threshold is associated with a high cortical excitability, a high resting motor threshold with a low cortical excitability. A recent review that investigated whether the rMT is a suitable biomarker for predicting post-stroke upper limb function found a correlation between rMT and upper limb motor function after stroke [18]. However, it needs still to be clarified how the rMT in either hemisphere changes in the course of motor recovery to identify potential mechanisms of functional restoration.

## Methods

### Data source

**Fig. 1:**
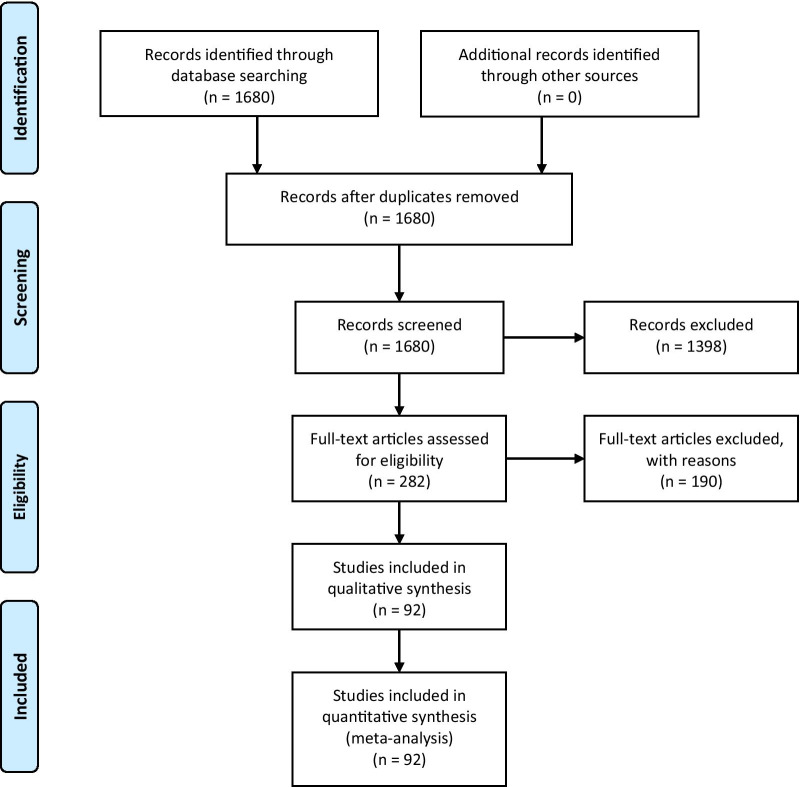
Summary of literature search results based on PRISMA guidelines

The PubMed research database was searched from its inception through to 31 October 2020 for studies investigating resting motor threshold as measured by TMS and motor function of both hands in stroke patients. The search terms “stroke”, “transcranial magnetic stimulation” and “motor” were used. The screening was performed by one reviewer. Figure 1 illustrates the actual search strategy.

### Study selection

Studies matching the following criteria were included: (1) study on humans, (2) diagnosis of stroke with the consequence of a hemiparesis/hemiplegia, (3) assessment of motor function of both the affected and the non-affected hand, (4) assessment of resting motor threshold of the ipsilesional and/or contralesional hemisphere, (5) prospective study and (6) more than four patients included. The appropriate studies were included, regardless of study design used (interventional—observational, crossover—longitudinal, different number of groups).

### Data extraction

The primary data extracted from the selected publications were (1) the hand motor function of the affected and non-affected hand and (2) the resting motor threshold of the affected and/or the non-affected hemisphere. For longitudinal studies, the baseline data, and the data of two last follow-up evaluations were extracted. If a healthy control group was available, the resting motor threshold of the non-dominant and the dominant hemisphere was extracted for a comparison with stroke subjects. The secondary selected data were (1) subjects characteristics (number, age, gender, time since stroke, stroke etiology and location), (2) methodological approach (study design, interventions, evaluations scheduling) and assessments (targeted muscle, stimulator and coil type, hand motor assessment) (Tables 1, 2, 3).

**Table 1:** Subjects characteristics and study design of studies included in the review

Patients characteristics			Study design								Healthy controls (number)	References
Number/sex/age (years)	Time since stroke	Stroke etiology and location	Cross-sectional	Longitudinal	Observational	Interventional	Interventions/groups	Evaluations schedule (days)				
								BL	1.FU	2.FU		
19/Na/69 ± 10	Acute phase	19 i/10sc, 9c/6r, 13 l	✘		✘			0				Nascimbeni et al. [60]
8 m, 8f/58 ± 18	< 1 day	19 i/19c/8r, 8 l		✘	✘		Na	0	365		20	Delvaux et al. [61]
7 m, 3f/37–80	1–5 days	19i/6sc, 4c/5r, 5 l	✘		✘		Na	0				Freundlieb et al. [62]
12 m, 9f/72 ± 3	1–5 days	16i, 5 h/6sc, 13c, 1na/7r, 14 l	✘		✘		Na	0				Trompetto et al. [37]
7 m, 5f/70 ± 10	5 ± 3 days	12i/3sc, 9c/3r, 9 l	✘		✘		Na	0			12	Di Lazzaro et al. [63]
8 m, 17f/53 ± 10	5 ± 3 days	25i/18sc, 8c/11r, 14 l	✘		✘		Na	0				Du et al. [27]
14 m, 6f/65 ± 11	5 ± 3 days	20i/sc,c/na		✘		✘	(1) Anodal tDCS	0	5	33		Sattler et al. [30]
							(2) Sham tDCS					
48 m, 12f/55 ± 11	5 ± 4 days	60i/46sc, 14c/28r, 33 l	✘		✘		Na	0				Du et al. [64]
15 m, 16f/64 ± 14	6 (1–18) days	31i/14sc, 17c/12r, 19 l	✘		✘		Na	0			29	Huynh et al. [28]
17 m, 9f/67 ± 13	7 ± 4 days	26i/21sc, 5c/22r, 4 l	✘		✘		Na	0				Volz et al. [65]
39 m, 44f/43–66	8 ± 2 days	83i/na/na		✘		✘	(1) CIMT	0	14			El Helow et al. [66]
							(2) CT					
10 m, 6f/69 ± 7	10 days	16i/na/10r, 6 l		✘		✘	(1) 1 Hz rTMS	0	45	90		Blesneag et al. [67]
							(2) Sham rTMS					
6/na/18–80	10 ± 3 days	6i/4sc, 2c/4r, 2 l		✘	✘		Na	0	90	180		Birchenall et al. [68]
6 m, 4f/58 ± 16	10 ± 4 days	10i/4sc, 6c/8r, 2 l		✘	✘		Na	0	30	180		Swayne et al. [34]
26 m, 14f/58 ± 9	13 ± 5 days	40i/14sc, 26c/22r, 18 l		✘		✘	(1) Anodal tDCS	0	7			Khedr et al. [69]
							(2) Cathodal tDCS					
							(3) Sham tDCS					
10 m, 8f/59 ± 3	< 4 weeks	18i/5sc, 13c/9r, 9 l	✘		✘		Na	0			13	Bütefisch et al. [70]
24 m, 7f/37 ± 8	14 days	31i/31sc/12r, 19 l		✘	✘		Na	0	14	28		Prashanta et al. [71]
13 m, 8f/60 ± 12	16 ± 5 days	9i, 12 h/na/16r, 5 l	✘		✘		Na	0				Lee et al. [72]
13 m, 4f/60 ± 10	25 days	na/8sc, 8c, 1na/11r, 6 l		✘		✘	(1) Virtual reality training	0	14	44		Yarossi et al. [73]
10 m, 12f/62 ± 14	27 ± 12 days	18i, 4 h/na/10r, 12 l		✘		✘	(1) 1 Hz rTMS + AO	0	10			Noh et al. [74]
							(2) 1 Hz rTMS					
16 m, 8f/64 ± 11	27 ± 7 days	10i, 14 h/24sc/na		✘	✘		Na	0	90	365	25	Takechi et al. [75]
6 m, 8f/68 ± 10	30 days	14i/7sc, 7c/5r, 7 l		✘	✘		Na	0	30			Lioumis et al. [76]
8 m, 2f/67 ± 7	30 ± 6 days	2i, 8 h/3sc, 7c/4r, 6 l	✘		✘		Na	0			10	Cincinelli et al. [77]
6 m, 4f/72 ± 8	30 ± 12 days	9i, 1 h/8sc, 2c/4r, 6 l	✘		✘		Na	0				Lüdemann-Podubecká et al. [78]
10 m, 7f/66 ± 15	31 ± 20 days	14i, 3 h/4sc, 13c/10r, 7 l	✘		✘		Na	0				Veldema et al. [38]
16 m, 12f/62 ± 14	32 ± 15 days	na/sc, c/16r, 12 l		✘	✘		Na	0	28			Platz et al. [79]
13 m, 7f/58 ± 11	34 ± 13 days	20i/11sc, 9c/8r, 12 l	✘		✘		Na	0				Renner et al. [80]
11 m, 4f/55 ± 18	34 ± 9 days	9i, 6 h/12sc, 3c/7r, 7 l, 1na	✘		✘		Na	0			15	Kim et al. [22]
6 m, 4f/60 ± 6	37 ± 15 days	10i/7sc, 3c/5r, 5 l		✘		✘	(1) TBS	0	1		10	Khan et al. [81]
							(2) NMES					
							(3) TBS + NMES					
11 m, 9f/64 ± 12	37 ± 17 days	15i, 5 h/11sc, 9c/11r, 9 l		✘	✘		Na	0	90	120		Traversa et al. [82]
9 m, 9f/62 ± 10	38 days	18i/18sc/14r, 4 l	✘		✘		Na	0			11	Renner et al. [83]
26 m, 14f/63 ± 9	40 ± 24 days	35i, 5 h/14sc, 26c/23r, 17 l	✘		✘		Na	0				Seniòw et al. [84]
6 m, 8f/67 ± 12	43 ± 12 days	11i, 3 h/6sc, 8c/6r, 8 l	✘		✘		Na	0			14	Brouwer et al. [25]
5 m, 4f/55 (42–68)	43 days	9i/7sc, 2c/3r, 6 l		✘		✘	(1) PT	0	0	1		Liepert et al. [85]
12 m, 6f/61 ± 12	35–60 days	na/10sc, 8c/3r, 16 l		✘	✘		Na	0	63		20	Cincinelli et al. [26]
2 m, 6f/76 ± 14	52 ± 37 days	4i, 4 h/4sc, 4c/3r, 5 l		✘	✘		Na	0	60			Matsura et al. [86]
9 m, 9f/70 ± 10	54 ± 44 days	na/8sc, 10c/11r, 7 l	✘		✘		Na	0				Veldema et al. [44]
16 m, 8f/50 ± 12	71 ± 39 days	15i, 8 h, 1ih/14sc, 10c/13r, 11 l	✘		✘		Na	0				Tarri et al. [87]
11 m, 6f/64 ± 10	73 ± 15 days	na/5sc, 12c/7r, 10 l	✘		✘		Na	0				Cincinelli et al. [88]
5 m, 3f/60 ± 13	83 ± 56 days	8i/8sc/2r, 6 l	✘		✘		Na	0			8	Liepert et al. [89]
6 m, 3f/62 ± 10	3 ± 1 months	8i, 1 h/5sc, 4c/7r, 2 l		✘	✘		Na	0	28		9	Grau-Sánchez et al. [90]
26 m, 14f/63 (57–71)	4 (1–59) months	40i, 24sc, 16c/20r, 20 l	✘		✘		Na	0			24	Kemlin et al. [91]
11 m, 9f/72 ± 13	< 6 months	20i/11sc, 9c/11r, 9 l	✘		✘		Na	0				Schambra et al. [92]
15 m, 6f/62 ± 9	> 6 months	21i/12sc, 9c/6r, 15 l	✘		✘		Na	0				
38 m, 10f/63 ± 12	5 ± 4 months	48i/22sc, 23c, 3na/na		✘		✘	(1) 1 Hz rTMS + iTBS	0	28	120		Wang et al. [45]
							(2) iTBS + 1 Hz rTMS					
							(3) sham					
13/na/58 ± 4	3–9 months	13i/na/na		✘	✘		Na	0	14	28		Sawaki et al. [123]
26/na/58 ± 4	> 3 months	26i/na/na		✘	✘		(1) 3–9 months since stroke	0	14	134		Sawaki et al. [93]
							(2) > 12 months since stroke					

Patients characteristics			Study design					Evaluations schedule (days)			Healthy controls (number)	References
Number/sex	Time since stroke	Stroke etiology and location	Cross-sectional	Longitudinal	Observational	Interventional	Groups	BL	1.FU	2.FU		
17 m, 7f/61 ± 13	6 ± 12 months	24i/10sc, 14c/17r, 7 l	✘		✘		Na	0				Theilig et al. [94]
26 m, 16f/59 ± 11	7 ± 6 months	na/na/28r, 14 l	✘		✘		Na	0				Chervyakov et al. [95]
41 m, 13f/63 ± 13	8 ± 2 months	35i, 19 h/35sc, 19c/na		✘		✘	(1) 1 Hz rTMS + iTBS (2) sham rTMS + iTBS (3) 1 Hz rTMS + sham iTBS (4) sham rTMS + sham iTBS	0	14	28		Sung et al. [96]
17 m, 9f/64 ± 12	6–18 months	Na/26sc/12r, 14 l	✘		✘		Na	0			20	Pennisi et al. [97]
36/na/66 ± 7	> 6 months	36i/sc, c/na	✘		✘		Na	0				Borich et al. [24]
5 m, 7f/26–75	14 ± 9 months	Na (6sc, 6c/6r, 6 l	✘		✘		Na	0				Bastings et al. [40]
13 m, 9f/64 ± 9	17 ± 7 months	22i/3sc, 19c/8r, 14 l	✘		✘		Na	0				Cakar et al. [23]
6 m, 4f/56 ± 11	17 ± 15 months	7i,3 h/na/4r, 6 l	✘		✘		Na	0				Shiner et al. [31]
6 m, 3f/52 ± 9	18 ± 6 months	9i/9sc/9 l	✘		✘		Na	0				Braun et al. [98]
6 m, 4f/61 ± 8	6–48 months	na/na/na	✘		✘		Na	0			10	Cruz Martínez et al. [99]
6 m, 1f/66 ± 9	23 ± 13 months	5i, 2 h/5sc, 2c/2r, 5 l		✘	✘		Na	0	21			Chouinard et al. [100]
7 m, 6f/69 ± 8	23 ± 16 months	10i, 3 h/13sc/4r, 9 l	✘		✘		Na	0				Ackerley et al. [101]
13 m, 7f/53 ± 14	27 ± 18 months	20i/na/8r, 12 l	✘		✘		Na	0				Takeuchi et al. [35]
10 m, 2f/57 ± 12	28 ± 30 months	12i/12sc/10r, 2 l	✘		✘		Na	0				Bestmann et al. [41]
15 m, 6f/54 ± 12	29 ± 38 months	na/8sc, 13c/9r, 12 l	✘		✘		Na	0				Stinear et al. [33]
7 m, 3f/59 ± 15	30 ± 25 months	na/na/6r, 4 l		✘		✘	(1) OT	0	28	58		Koski et al. [21]
15 m, 5f/59 ± 9	6–74 months	na/12sc, 8c/9r, 11 l		✘	✘		Na	0	28			Amangual et al. [124]
2 m, 4f/58 ± 15	31 ± 38 months	6i/3sc, 3c/1r, 5 l	✘		✘		Na	0				Talelli et al. [102]
13 m, 3f/64 (41–81)	33 (12–86) months	16i/na/2r, 14 l		✘		✘	(1) CIMT (2) control	0	10			Wittenberg et al. [103]
12 m, 8f/61 ± 6	38 ± 38 months	Na/na/6r, 14 l	✘		✘		Na	0				Milot et al. [104]
15 m, 3f/67 ± 2	40 ± 6 months	Na/12sc, 6c/5r, 13 l	✘		✘		Na	0			17	Guder et al. [42]
7 m, 5f/60 ± 11	40 ± 25 months	Na/6sc, 6c/4r, 8 l		✘	✘		Na	0	12			Liepert et al. [105]
17 m, 13f/65 ± 9	40 ± 27 months	Na/8sc, 21c/17r, 13 l		✘		✘	(1) PT (2) RMV + PT	0	7	14		Marconi et al. [106]
17 m, 6f/56 ± 14	43 ± 63 months	23i/23sc/15r, 8 l	✘		✘		Na	0				Thickbroom et al. [36]
50 m, 32f/68 (42–90)	43 (5–227) months	82i/na /82 l		✘	✘		Na	0	90	270		Edwards et al. [20]
6 m, 3f/40 ± 5	44 ± 8 months	9i/2sc, 7c/1r, 8 l	✘		✘		Na	0				Conforto et al. [107]
11 m, 8f/66 ± 11	45 ± 36 months	19i/13sc, 6c/11r, 8 l	✘		✘		Na	0				Palmer et al. [16]
4 m, 5f/57 ± 17	50 ± 41 months	9i/sc, c/5r, 4 l	✘		✘		Na	0				Von Lewinski et al. [108]
8 m, 4f/62 ± 10	51 ± 30 months	12i/12c/6r, 6 l	✘		✘		Na	0				Carey et al. [109]
8 m, 5f/66 ± 10	52 ± 39 months	13i/13sc/7r, 6 l	✘		✘		Na	0			12	Gray et al. [110]
50 m, 20f/60 ± 12	57 ± 63 months	i, h/na/38r, 32 l	✘		✘		Na	0				Kuppuswamy et al. [111]
8 m, 3f/66 ± 9	57 ± 52 months	9i, 2 h/5sc, 6c/3r, 8 l	✘		✘		Na	0				Cassidy et al. [112]
4 m, 6f/62 ± 12	4.8 ± 5.5 years	10i/7sc, 3c/6r, 4 l		✘	✘		Na	0	14	28		Restemeyer et al. [113]
11 m, 1f/71 ± 9	5.0 ± 4.4 years	na/1sc, 11c/7r, 5r	✘		✘		Na	0			16	Mooney et al. [114]
10 m, 8f/62 ± 12	5.1 ± 1.0 years	18i/10sc, 8c/8r, 10 l	✘		✘		Na	0			18	Buetefisch et al. [115]
10 m, 4f/48–91	5.1 ± 3.8 years	14i/na/na	✘		✘		Na	0				Silverstein et al. [32]
17 m, 10f/61 ± 8	5.6 ± 4.0 years	na/27sc/14r, 13 l	✘		✘		Na	0				Takeuchi et al. [116]
10 m, 3f/57 ± 10	5.9 ± 4.7 years	13i/10sc, 3c/2r, 11 l		✘	✘		Na	0	40	194		Liepert et al. [117]
19 m, 5f/65 ± 9	6.0 ± 5.1 years	na/14sc, 8c/na	✘		✘		Na	0			11	Mang et al. [43]
14 m, 6f/62 ± 14	6.2 ± 2.7 years	20i/13sc, 7c/12r, 8 l	✘		✘		Na	0				Werhahn et al. [39]
9 m, 4f/66 ± 9	6.2 ± 3.6 years	na/8sc, 4c/6r, 7 l	✘		✘		Na	0				Miller et al. [29]
26 m, 6f/61 ± 8	6.8 ± 3.5 years	20i, 12 h/na/12r, 20 l	✘		✘		Na	0				Liu et al. [118]
6 m, 2f/59 ± 9	7.3 ± 7.5 years	na/6sc, 2c/4r, 4 l		✘	✘		Na	0	28			Liepert et al. [119]
22 m, 5f/61 ± 8	7.6 ± 2.3 years	17i, 10 h/na/9r, 18 l	✘		✘		Na	0			15	Liu et al. [120]
6 m, 8f/62 ± 16	8.0 ± 11.3 years	9i, 5 h/7sc, 7c/8r, 6 l	✘		✘		Na	0			14	Brouwer et al. [25]
11 m, 5f/59 ± 10	10.0 ± 6.0 years	na/na/6r, 10 l	✘		✘		Na	0			9	Lewis et al. [121]
5 m, 5f/16 ± 6	16 ± 6 years	na /sc, c/4r, 6 l	✘		✘		Na	0			8	Berweck et al. [122]

AO, action observation; c, cortical involvement; BL, baseline; CIMT, constraint induced movement therapy; f, female; h, hemorrhagic; Hz, hertz; I, ischemic; iTBS, intermittent theta burst stimulation; l, left; m, male; na, not available, not applicable; NMES, neuromuscular electrical stimulation; OT, occupational therapy; PT, physiotherapy; r, right; RMV, repeated muscle vibration; rTMS, repetitive transcranial magnetic stimulation; sc, subcortical; tDCS, transcranial direct current stimulation; (i)TBS, (intermittent) theta burst stimulation; 1. FU, first follow-up; 2. FU, second follow-up

**Table 2:** Motor function of the affected and the non-affected hand (means) and laterality quotients (means) of studies included in the review

Test (units)	Interventions/groups	Baseline			1. Follow up			2. Follow up			1. Follow up—baseline changes			2. Follow up—baseline changes			References
		NA	A	LQ	NA	A	LQ	NA	A	LQ	NA	A	LQ	NA	A	LQ	
MI (UL) (score)	Na	100	10	82													Nascimbeni et al. [60]
MRC (score)	Na	5.0	0.7	75	5.0	4.0	11				0.0	3.3	−64				Delvaux et al. [61]
FM (score)	Na	66	44	20													Freundlieb et al. [62]
SSS hand (score)	Na	6.0	1.1	69													Trompetto et al. [37]
NIHSS (UL) (score)	Na	0.0	1.9	100													Di Lazzaro et al. [63]
FM (score)	Na	66	34	32													Du et al. [27]
FM (score)	(1) Anodal tdcs	66	47	17	66	54	10	66	60	5	0	7	−7	0	13	−12	Sattler et al. [30]
	(2) Sham tdcs	66	49	15	66	58	6	66	61	4	0	9	−8	0	12	−11	
FM (score)	Na	66	28	40													Du et al. [64]
FM (score)	Na	66	50	14													Huynh et al. [28]
Grip strength (N)	Na	65	25	44													Volz et al. [65]
FM (score)	(1) CT	66	36	29	66	37	28				0	1	−1				El Helow et al. [66]
	(2) CIMT	66	32	35	66	50	14				0	18	−21				
FM (score)	(1) Sham rTMS	66	32	35	66	38	27	66	42	22	0	6	−8	0	10	−12	Blesneag et al. [67]
	(2) 1 Hz rTMS	66	29	39	66	43	21	66	45	19	0	14	−18	0	16	−20	
FM (score)	Na	66	15	63	66	50	14	66	46	18	0	35	−49	0	31	−45	Birchenall et al. [68]
ARAT (score)	Na	57	34	25	57	45	12	57	51	6	0	11	−14	0	17	−20	Swayne et al. [34]
MRC (score)	(1) Anodal tdcs	5.0	1.5	54	5.0	3.3	20				0.0	1.8	−33				Khedr et al. [69]
	(2) Cathodal tdcs	5.0	2.0	43	5.0	3.4	19				0.0	1.4	−24				
	(3) Sham tdcs	5.0	1.5	54	5.0	2.4	35				0.0	0.9	−19				
MI (UL) (score)	Na	100	63	23													Bütefisch et al. [70]
MRC (score)	Na	5.0	0.0	100	5.0	2.7	30	5.0	2.6	32	0.0	2.7	−70	0.0	2.6	−68	Prashanta et al. [71]
FM (score)	Na	66	23	48													Lee et al. [72]
FM (score)	(1) VRT	66	24	47	66	35	31	66	46	18	0	11	−16	0	22	−29	Yarossi et al. [73]
FM (score)	(1) 1 Hz rTMS + AO	66	28	40	66	40	25				0	12	−16				Noh et al. [74]
	(2) 1 Hz rTMS	66	21	52	66	31	36				0	10	−16				
FM (score)	Na	66	30	38	66	44	20	66	45	19	0	14	−18	0	15	−19	Takechi et al. [75]
ARAT (score)	Na	57	48	9	57	50	7				0	2	−2				Lioumis et al. [76]
CNS (UL) (score)	Na	4.5	1.1	61													Cincinelli et al. [77]
JTHFT (sec)	Na	6.39	10.41	24													Lüdemann-Podubecká et al. [46]
WMFT (score)	Na	70	32	37													Veldema et al. [44]
FM (score)	Na	66	24	47	66	36	29				0	12	−17				Platz et al. [79]
MI (UL) (score)	Na	100	62	23													Renner et al. [80]
FM (score)	Na	66	55	9													Kim et al. [22]
NHPT (time)	(1) TBS	36	22	24	34	22	21				−2	0	−3				Khan et al. [81]
	(2) NMES	36	22	24	34	21	24				−2	−1	−1				
	(3) TBS + NMES	36	21	26	30	21	18				−6	0	−9				
CNS (UL) (score)	Na	4.5	0.5	80	4.5	0.9	67	4.5	1.0	64	0.0	0.4	−13	0.0	0.5	−16	Traversa et al. [82]
RMA (score)	Na	15.0	11.8	12													Renner et al. [83]
WMFT (score)	Na	75	38	33	75	48	22	75	55	15	0	10	−11	0	17	−17	Seniòw [84]
MAS (UL) (score)	Na	68	24	48													Brouwer et al. [25]
FAT (score)	(1) PT	5	3.8	14	5	3.8	14	5	3.8	14	0	0	0	0	0.0	0	Liepert et al. [119]
CNS (UL) (score)	Na	4.50	0.36	85													Cincinelli et al. [26]
FM (score)	Na	66	52	12	66	58	6				0	6	−5				Matsura et al. [86]
WMFT (score)	Na	70	31	39													Veldema et al. [44]
FM (score)	Na	66	23	48													Tarri et al. [87]
MI (UL) (score)	Na	100	72	16													Cincinelli et al. [88]
Grip strength (N)	Na	75	62	9													Liepert et al. [89]
ARAT (score)	Na	57	38	20	57	46	11				0	8	−9				Grau-Sánchez et al. [90]
FM (score)	Na	66	52	12													Kemlin et al. [91]
MRC (score)	(1) < 6 months	5.0	4.4	6													Schambra et al. [92]
	(2) > 6 months	5.0	4.0	11													
WMFT (score)	(1) 1 Hz rTMS + iTBS	75	30	43	75	38	33	75	40	30	0	8	−10	0	10	−12	Wang et al. [45]
	(2) iTBS + 1 Hz rTMS	75	31	42	75	35	36	75	37	34	0	4	−5	0	6	−8	
	(3) sham	75	31	42	75	32	40	75	31	42	0	1	−1	0	0	0	
WMFT (sec)	Na	0.48	1.30	46	0.43	1.20	47	0.45	1.26	47	−0.05	−0.10	1	−0.03	−0.04	1	Sawaki et al. [123]
WMFT (min)	(1) 3–9 months	0.36	1.17	53	0.32	0.88	47	0.42	0.96	39	−0.04	−0.29	−6	0.06	−0.21	−14	Sawaki et al. [93]
	(2) > 12 months	0.42	1.24	49	0.39	1.18	50	0.31	1.21	59	−0.03	−0.06	1	−0.11	−0.03	10	
WMFT (score)	Na	75	15	67													Theilig et al. [94]
FM (score)	Na	66	32	35													Chervyakov et al. [95]
WMFT (score)	(1) 1 Hz rTMS + iTBS	75	31	42	75	33	39	75	39	32	0	2	−3	0	8	−10	Sung et al. [96]
WMFT (score)	(2) Sham rTMS + iTBS	75	31	42	75	31	42	75	33	39	0	0	0	0	2	−3	
WMFT (score)	(3) 1 Hz rTMS + sham iTBS	75	33	39	75	40	30	75	35	36	0	7	−8	0	2	−3	
WMFT (score) (sham + sham)	(4) Sham rTMS + sham iTBS	75	31	42	75	31	42	75	32	40	0	0	0	0	1	−1	
NHPT (time)	Na	17	19	3													Pennisi et al. [97]
B&B (score)	Na	55	29	31													Borich et al. [24]
MI (UL) (score)	Na	33	25	14													Bastings et al. [40]
MI (UL) (score)	Na	100	57	27													Cakar et al. [23]
FM (score)	Na	66	55	9													Shiner et al. [31]
MRC (score)	Na	5.0	4.2	9													Braun et al. [98]
CNS (UL) (score)	Na	4.5	2.2	34													Cruz-Martínez et al. [99]
MAL (score)	Na	5.0	2.6	32	5.0	3.9	12				0.0	1.3	−19				Chouinard et al. [100]
FM (score)	Na	66	41	23													Ackerley et al. [101]
FM (score)	Na	66	45	19													Takeuchi et al. [35]
ARAT (score)	Na	57	48	9													Bestmann et al. [41]
FM (score)	Na	66	16	61													Stinear et al. [33]
FM (score)	Na	66	36	29	66	42	22	66	45	19	0	6	−7	0	9	−10	Koski et al. [21]
ARAT (score)	Na	57	42	15	57	47	10				0	4	−5				Amengual et al. [124]
ARAT (score)	Na	57	51	6													Talelli et al. [102]
MAL (score)	(1) CIMT	5.0	1.1	64	5.0	2.2	39				0.0	1.1	−25				Wittenberg et al. [103]
	(2) Control	5.0	1.3	59	5.0	1.3	59				0.0	0.0	0				
FM (score)	Na	66	52	12													Milot et al. [104]
FM (score)	Na	66	57	7													Guder et al. [42]
MAL (score)	Na	5.0	2.2	39	5.0	3.0	25				0.0	0.8	−14				Liepert et al. [105]
WMFT (score)	(1) PT	75	40	30	75	42	28	75	43	27	0	2	−2	0	3	−3	Marconi et al. [106]
	(2) RMV + PT	75	38	33	75	46	24	75	53	17	0	8	-9	0	15	−16	
Grip strength (N)	Na	40	37	3													Thickbroom et al. [36]
FM (score)	Na	66	25	45	66	33	33	66	34	32	0	8	−12	0	9	−13	Edwards et al. [20]
FM (score)	Na	66	63	2													Conforto et al. [107]
FM (score)	Na	66	50	14													Palmer et al. [16]
ARAT (score)	Na	57	8	75													Von Lewinski et al. [108]
FM (score)	Na	66	39	26													Carey et al. [109]
FM (score)	Na	66	51	13													Gray et al. [110]
ARAT (score)	Na	57	56	1													Kuppuswamy et al. [111]
FM (score)	Na	66	46	18													Cassidy et al. [112]
ARAT (score)	Na	57	54	3	57	54	3	57	54	3	0	0	0	0	0	0	Restemeyer et al. [113]
FM (score)	Na	66	48	16													Mooney et al. [114]
JTHFT (min)	Na	0.16	0.49	51													Buetefisch et al. [115]
FM (score)	Na	66	33	33													Silverstein et al. [32]
FM (score)	Na	66	52	12													Takeuchi et al. [116]
MAL (score)	Na	5.0	2.2	39	5.0	3.7	15				0.0	1.5	−24				Liepert et al. [117]
FM (score)	Na	66	41	23													Mang et al. [43]
FM (score)	Na	66	44	20													Werhahn et al. [39]
FM (score)	Na	66	30	38													Miller et al. [29]
FM (score)	Na	66	58	6													Liu et al. [118]
MAL (score)	Na	5.0	2.3	37	5.0	3.0	25				0.0	0.7	−12				Liepert et al. [119]
FM (score)	Na	66	59	6													Liu et al. [120]
MAS (UL) (score)	Na	68	46	19													Brouwer et al. [25]
FM (score)	Na	60	31	32													Lewis et al. [121]
MACS (score)	Na	1.0	1.8	29													Berweck et al. [122]

A, affected hand; AO, action observation; ARAT, Action Research Arm Test; B&B, Box and Block Test; CIMT, constraint induced movement therapy; CNS, Canadian Neurological Scale; CT, conventional treatment; FAT, Frenchay Arm Test; FM, Flugl Meyer assessment; Hz, hertz; iTBS, intermittent theta burst stimulation; JTHFT, Jebsen Taylor Hand Function Test; LQ, laterality quotient; MACS, Manual Ability Classification System; MAL, Motor Activity Log; MAS, Motor Assessment Scale; MI, Motoricity Index; min, minute; MRC, British Medical Research Council; N, Newton; NA, non affected hand; NHPT, Nine Hole Peg Test; NIHSS, National Institutes of Helth Stroke Scale; NMES, neuromuscular electrical stimulation; PT, physiotherapy; RMA, Rivermead Motor Assessment; RMV, repeated muscle vibration; rTMS, repetitive transcranial magnetic stimulation; sec, second; SSS, Scandinavian Stroke Scale; TBS, theta burst stimulation; tDCS, transcranial direct current stimulation; UL, upper limb; VRT, virtual reality training; WMFT, Wolf Motor Function Test

**Table 3:** Resting motor threshold of the affected and the non-affected hemisphere (means) and laterality quotients (means) of studies included in the review

Targeted muscle, stimulator type, coil type	Interventions/groups	Baseline			1. Follow up			2. Follow up			1. Follow up—baseline changes			2. Follow up—baseline changes			Healthy controls			References
		NA	A	LQ	NA	A	LQ	NA	A	LQ	NA	A	LQ	NA	A	LQ	D	ND	LQ	
FDI, MS-200, RC	Na		95																	Nascimbeni et al. [60]
FDI, MS-200, F8C	Na	55	68	−11	50	56	−6				−5	−12	5				48	48	0	Delvaux et al. [61]
FDM, NS-eX, na	Na	38	55	−18																Freundlieb et al. [62]
TM, MS-200, RC	Na	57	79	−16																Trompetto et al. [37]
FDI, MS-200, F8C	Na	56	68	−10													55	54	1	Di Lazzaro et al. [63]
APB, MP-X100, F8C	Na	47	68	−18																Du et al. [27]
ECR, MS-200, F8C	(1) Anodal tDCS		70			66			65			−4			−5					Sattler et al. [30]
	(2) Sham tDCS		62			57			56			−5			−6					
APB, MP-X100, F8C	Na		73																	Du et al. [64]
APB, MS-BS, RC	Na	58	66	−6													62			Huynh et al. [28]
APB, MS-200, F8C	Na		67																	Volz et al. [65]
APB, ML-200, F8C	(1) CT		70			68						–2								El Helow et al. [66]
	(2) CIMT		70			61						−9								
APB, MP-X100, F8C	(1) Sham rTMS	62	74	−9	68	66	1	72	67	4	6	−8	10	10	−7	12				Blesneag et al. [67]
	(2) 1 Hz rTMS	53	63	−9	79	66	9	70	68	1	26	3	18	17	5	10				
FDI, MS-200, F8C	Na	51	93	−29	50	85	−26	48	76	−23	−1	−8	3	−3	−17	7				Birchenall et al. [68]
FDI, MS-BS, F8C	Na	42	64	−21	43	57	−14	45	57	−12	1	−7	7	3	−7	9				Swayne et al. [34]
FDI, MS-200, F8C	(1) Anodal tDCS	37	60	−24	37	48	−13				0	−12	11							Khedr et al. [69]
	(2) Cathodal tDCS	34	60	−28	33	51	−21				−1	−9	6							
	(3) Sham tDCS	35	63	−29	34	59	−27				−1	−4	2							
FDI, MS-BS, F8C	Na	59															51	51	0	Bütefisch et al.[70]
FDI, MS-200, F8C	Na	44	95	−37	39	93	−41	37	93	−43	−5	−2	−4	−7	−2	−6	42			Prashanta et al. [71]
FDI, MS-R, F8C	Na	79																		Lee et al. [72]
FDI, MS-200, F8C	(1) VRT	60	91	−21	59	90	−21	62	90	−18	−1	−1	0	2	−1	2				Yarossi et al. [73]
APB, MP-X100, F8C	(1) 1 Hz rTMS + AO	70			71						1									Noh et al. [74]
	(2) 1 Hz rTMS	67			73						6									
FDI, MS-BS, F8C	Na	47	74	−22	51	66	−13	52	59	−6	4	−8	9	5	−15	16	54			Takechi et al. [75]
FDI, NS-eX, F8C	Na	57	69	−10	57	67	−8				0	−2	1							Lioumis et al. [76]
ADM, MS-BS, F8C	Na	59	69	−8													59	59	0	Cincinelli et al. [77]
FDI, MS-RS, F8C	Na	60	66	−5																Lüdemann-Podubecká et al. [78]
FDI, MS-RS, F8C	Na	60	92	−21																Veldema et al. [38]
APB, MS-200, F8C	Na	55	89	−24	54	83	−21				−1	−6	2							Platz et al. [79]
FCU, MS-BS, F8C	Na		64																	Renner et al. [80]
ECR, MP-R30, RC	Na		53															51		Kim et al. [22]
FDI, MS-R, F8C	(1) TBS	54	54	0	55	50	5				1	−4	5				54	54	0	Khan et al. [81]
	(2) NMES	54	54	0	54	49	5				0	−5	5							
	(3) TBS + NMES	50	54	−4	54	43	11				4	−11	15							
ADM, MS-200, RC	Na	48	70	−19	47	63	−15	45	61	−15	−1	−7	4	−3	−9	4				Traversa et al. [82]
FDI, MS-200, F8C	Na		62															43		Renner et al. [83]
FDI, MS-R, F8C	Na	60																		Seniòw et al. [84]
FDI, na, F8C	Na	63	86	−15													58	57	1	Brouwer et al. [25]
APB, MS-na, F8C	(1) PT	45	55	−10	45	57	−12	45	57	−12	0	2	−2	0	2	−2				Liepert et al. [119]
ADM, MS-R, F8C	Na	42	71	−26													47	48	−1	Cincinelli et al. [26]
FCR, MS-200, F8C	Na	50			50						0									Matsura et al. [86]
APB, MS-SR, F8C	Na	64	86	−15																Veldema et al. [44]
ECR, MP-na, F8C	Na		71																	Tarri et al. [87]
ADM, MS-R, F8C	Na	48	61	−12																Cincinelli et al. [88]
FDI, MS-200, F8C	Na	45	45	0													42			Liepert et al. [89]
FDI, MS-R, F8C	Na	54	73	−15	56	67	−9				2	−6	6				67	70	−2	Grau-Sánchez et al. [90]
FDI, MS-200, F8C	Na	42	56	−14													41			Kemlin et al. [91]
FDI, MS-BS, F8C	(1) < 6 months	47	51	−4													49	50	−1	Schambra et al. [92]
	(2) > 6 months	46	56	−10																
FDI, MS-200, F8C	(1) 1 Hz rTMS + iTBS	72	85	−8	74	78	−3	74	80	−4	2	−7	6	2	−5	4				Wang et al. [45]
	(2) iTBS + 1 Hz rTMS	71	78	−5	74	75	−1	76	76	0	3	−3	4	5	−2	5				
	(3) Sham	75	88	−8	74	85	−7	72	86	−9	−1	−3	1	−3	−2	−1				
EDC, MS-200, F8C	Na	47	61	−12	47	59	−11	49	66	−15	0	−2	1	2	5	−2				Sawaki et al. [123]
EDC, MS-200, F8C	(1) 3–9 months	52	65	−11	51	63	−10	52	62	−8	−1	−2	1	0	−3	2				Sawaki et al. [93]
	(2) > 12 months	44	55	−11	45	63	−17	46	53	−7	1	8	−6	1	−3	4				
ECR, MS-SR, F8C	Na	56																		Theilig et al. [94]

Targeted muscle	Interventions/groups	Baseline			1. Follw up			2. Follw up			1. Follw up—baseline changes			2. Follw up—baseline changes			Healthy controls			References
		NA	A	LQ	NA	A	LQ	NA	A	LQ	NA	A	LQ	NA	A	LQ	D	ND	LQ	
APB, MS-R, F8C	Na	47	80	−26																Chervyakov et al. [95]
FDI, MS-200, F8C	(1) 1 Hz rTMS + iTBS	70	86	−10	72	80	−5	74	77	−2	2	−6	5	4	−9	8				Sung et al. [96]
	(2) Sham rTMS + iTBS	71	87	−10	71	88	−11	74	85	−7	0	1	−1	3	−2	3				
	(3) 1 Hz rTMS + sham iTBS	71	84	−8	72	80	−5	70	81	−7	1	−4	3	−1	−3	1				
	(4) Sham rTMS + sham iTBS	70	85	−10	71	86	−10	70	84	−9	1	1	0	0	−1	1				
FDI, MS-NM-200, RC	Na	41	45	−4													43			Pennisi et al. [97]
ECR, MS-200, F8C	Na	43	48	−5																Borich et al. [24]
FDI, MS-200, F8C	Na	67	77	−7																Bastings et al. [40]
ADM, MP-X100, PC	Na	37	50	−15																Cakar et al. [23]
FDI, MS-200, RC	Na	46	73	−23																Shiner et al. [31]
APB, MS-200, F8C	Na	47	44	3																Braun et al. [98]
Na, na, na	Na	53	63	−9													55			Cruz-Martínez et al. [99]
Hand, C-HS, RC	Na	65	69	−3	66	70	−3				1	1	0							Chouinard et al. [100]
FDI, MS-200, F8C	Na	45	67	−20																Ackerley et al. [101]
FDI, MS-200, F8C	Na		65																	Takeuchi et al. [35]
FDI, MS-200, F8C	Na	44	57	−13																Bestmann et al. [41]
ECR, MS-200, F8C	Na	48	85	−28																Stinear et al. [33]
FDI, APB, ADM, C-HS/MS-R, F8C	Na	73	88	−9	69	82	−9	67	81	−9	−4	−6	1	−6	−7	0				Koski et al. [21]
FDI, MS-R, F8C	Na		66			63						−3								Amengual et al. [124]
ADM, MS-200, F8C	Na	39	47	−9																Talelli et al. [102]
EDC, MS-BS, F8C	(1) CIMT	50	71	−17	48	73	−21				−2	2	−3							Wittenberg et al. [103]
	(2) Control	50	72	−18	55	82	−20				5	10	−2							
ECR, MS-200, F8C	Na		59																	Milot et al. [104]
FDI, MS-200, F8C	Na	42	44	−2													37	36	1	Guder et al. [42]
FDI, MS-200, F8C	Na	43	46	−3	43	45	−2				0	−1	1							Liepert et al. [105]
FRC, BS-200, F8C	(1) PT		67			68			69			1			2					Marconi et al. [106]
	(2) RMV + PT		70			58			60			−12			−10					
BB, BS-200, F8C	(1) PT		72			70			68			−2			−4					
	(2) RMV + PT		72			59			62			−13			−10					
EDC, BS-200, F8C	(1) PT		68			67			71			−1			3					
	nnnnnn RMV + PT		70			60			64			−10			−6					
FDI, MS-200, F8C	Na	47	51	−4																Thickbroom et al. [36]
FCR, MP-X100, F8C	Na		84			81			80											Edwards et al. [20]
APB, MS-BS, F8C	Na	53																		Conforto et al. [107]
APB, MS-200, F8C	Na	53	60	−6																Palmer et al. [16]
APB, MS-200, F8C	Na	38	78	−34																Von Lewinski et al. [108]
ED, MS-R, F8C-AF	Na		52																	Carey et al. [109]
APB, MS-200, F8C	Na	51	55	−4													53	46	7	Gray et al. [110]
FDI, MS-BS, F8C	Na		53																	Kuppuswamy et al. [111]
FDI, MS-BS, F8C	Na	44	56	−12																Cassidy et al. [112]
FDI, na, F8C	Na	55	61	−5	54	61	−6	54	61	−6	−1	0	−1	−1	0	−1				Restemeyer et al. [113]
FDI, MS-BS, F8C	Na	48	47	1													51			Mooney et al. [114]
ECU, MS-BS, F8C	Na		67														53			Buetefisch et al. [115]
FDI, MS-BS, F8C	Na	46	76	−25																Silverstein et al. [32]
FDI, MS-200, F8C	Na	52	60	−7																Takeuchi et al. [116]
APB, MS-na, F8C	Na	46	55	−10	45	55	−10				−1	−1	0							Liepert et al. [117]
ECR, MS-200, F8C	Na	46	72	−22													45	46	−1	Mang et al. [43]
FDI, MS-200, F8C	Na	39	62	−23																Werhahn et al. [39]
ECR, MS-SR-F8C	Na		87																	Miller et al. [29]
FDI, MS-na, F8C	Na	44	50	−6																Liu et al. [118]
]FDI, MS-na, F8C	Na		51			51						0								Liepert et al. [119]
FDI, MS-na, F8C	Na	46	49	−3														46		Liu et al. [118]
FDI, na, F8C	Na	63	76	−9													58	57	1	Brouwer et al. [25]
APB, MS-BS, F8C	Na	52															42	46	−5	Lewis et al. [121]
FPB, MS-BS, F8C	Na	54	76	−17													46	43	3	Berweck et al. [122]

A, affected hemisphere; ADM, abductor digiti minimi; AO, action observation; APB, abductor pollicis brevis; BB, musculus biceps brachii; C-HS, Cadwell high-speed stimulator; CIMT, constraint induced movement therapy; CT, conventional; D, dominant hemisphere; ECR, extensor carpi radialis; ECU, extensor carpi ulnaris; ED, extensor digitorum; EDC, extensor digitorum communis; FDI, first dorsal interosseous muscle; FDM, flexor digiti minimi; Hz, hertz; iTBS, intermittent theta burst stimulation; FCU, flexor carpi ulnaris; FPB, flexor pollicis brevis; FCR, flexor carpi radialis; F8C, figure-of-eight shaped coil; LQ, laterality quotient; MEP, motor evoked potentials; ML-200, Maglit—200 stimulator; MP-X100, Magpro—X100 stimulator; MP-R30, Magpro—R30 stimulator; MS-BS, Magstim—BiStim stimulator; MS-NM-200, Magstim—Novametric—2000; MS-SR, Magstim—Super Rapid stimulator; MS-R, Magstim—Rapid stimulator; MS-200, Magstim—200 stimulator; na, not available, not applicable; NA, non affected hemisphere; NDH, non-dominant hemisphere; NMES, neuromuscular electrical stimulation; NS-eX, Nexstim—eXimia stimulator; PC, parabolic coil; PT, physiotherapy; RC, round coil; rMT, resting motor threshold; rMV, repeated muscle vibration; rTMS, repetitive transcranial magnetic stimulation; tDCS, transcranial direct current stimulation; TM, thenar muscles

### Hand motor function

Table 2 summarizes data on hand motor function of the affected and the non-affected hand. In case a study provided more than one hand motor function assessment, we selected those involving motor activities of daily living (e.g., Flugl-Meyer assessment or Wolf Motor Function Test) for our analysis. To account for differences in hand motor assessments used across studies, a laterality quotient was calculated for each study. The laterality quotient was calculated as follows: $\left( {\frac{Non - affected\,Hand - affected\,Hand}{{Non - affected\,Hand + affected\,Hand}}} \right)*100$. The laterality quotient varies between 0 and ± 100. The greater the laterality differences, the stronger the hand motor disability. Depending on the test used, motor impairment of the affected hand is associated with either a positive (e.g., Wolf Motor Function Test) or a negative value (e.g., Nine Hole Peg Test). To account for these differences, absolute values of the laterality quotient were used for the analysis of hand motor function.

### Resting motor threshold

Table 3 summarizes data on resting motor threshold of the ipsilesional (non-dominant) and the contralesional (dominant) hemisphere. If the MEP was not evocable, rMT was set to 100. If data for both hemispheres were available, we calculated laterality quotients for the resting motor threshold as: $\left( {\frac{Contralesional\, hemisphere - ipsilesional\, hemisphere}{{contralesional\, hemisphere + ipsilesional\, hemisphere}}} \right)*100$ for stroke patients, and as: $\left( {\frac{Dominant \,hemisphere - non - dominant\, hemsiphere}{{Dominant \,hemisphere + non - dominant \,hemisphere}}} \right)*100$ for healthy controls. Negative values are associated with a between-hemispheric imbalance towards the contralesional (dominant) hemisphere, positive values with a between-hemispheric imbalance towards the lesioned (non-dominant) hemisphere.

### Data synthesis and statistical analysis

Data was analyzed using SPSS Statistic 21 (IBM Corporation, USA). “Post–pre” differences between baseline and first follow-up, as well as baseline and second follow up were calculated for longitudinal data of stroke patients. Furthermore, differences between rMT in stroke patients and healthy controls were calculated for studies that included healthy control group. Pearson correlations were calculated between:

The amount of the hand motor impairment (expressed as laterality quotient of the hand motor function) and
  - The ipsilesional resting motor threshold,
  - The contralesional resting motor threshold,
  - the between-hemispheric imbalance of resting motor threshold (expressed as laterality quotient of the resting motor thresholds).
The amount of hand motor recovery (expressed as baseline—follow-up changes of laterality quotient of the hand motor function) and
  - The baseline—follow-ups changes of the ipsilesional resting motor threshold,
  - The baseline—follow-ups changes of the contralesional resting motor threshold,
  - The baseline—follow-ups changes of the between-hemispheric imbalance of resting motor thresholds.
(a) The ipsilesional resting motor threshold, (b) the contralesional resting motor threshold and (c) the between-hemispheric imbalance of resting motor thresholds.

R-Values ≥ 0.3 and p-values ≤ 0.05 are considered to be statistically relevant [19].

## Results

We identified 92 studies that matched our inclusion criteria. The studies show large variability of methods, participants, and results.

### Methods

#### Study design

57 studies have a cross-sectional observational study design. The remaining 35 studies were either observational (21 studies) or interventional randomized (10 studies) or interventional non-randomized (4 studies) longitudinal trials that investigated hand motor function and cortical excitability for up to one year. 25 studies included a healthy control group for resting motor threshold comparison. Table 1 illustrates study design of studies enrolled.

#### Hand motor function assessments

Overall 17 different hand motor assessments were used: Action Research Arm Test, Box and Block Test, Canadian Neurological Scale, Frenchay Arm Test, Flugl Meyer assessment, Grip strength, Jebsen Taylor Hand Function Test, Manual Ability Classification System, Motor Activity Log, Motor Assessment Scale, Motoricity Index, British Medical Research Council, Nine Hole Peg Test, National Institutes of Helth Stroke Scale, Rivermead Motor Assessment, Scandinavian Stroke Scale, Wolf Motor Function Test. Table 2 shows the overview of hand motor assessments applied.

#### Resting motor threshold assessments

13 different upper limb muscles were targeted to investigate resting motor threshold: abductor digiti minimi, abductor pollicis brevis, musculus biceps brachii, extensor carpi radialis, extensor carpi ulnaris, extensor digitorum, extensor digitorum communis, first dorsal interosseous muscle, flexor digiti minimi, flexor carpi ulnaris, flexor pollicis brevis, flexor carpi radialis, thenear muscles. Two studies did not specify which upper limb muscle has been targeted. Ten different stimulator types from five different manufacturers were used: Magstim 200, Magstim BiStim 200, Magstim Rapid, Magstim Super Rapid, Magstim Novamentric 2000 (MagStim Co., Withland, Dyfed, UK), Magpro X100, Magpro R30 (Mag Venture, Farum, Denmark), Maglit 200 (Dantec Dynamics Ltd, Bristol, UK), Nexstim eXimia (Nexstim Ltd, Helsinki, Finland) and Cadwell high-speed magnetic stimulator (Cadwell Inc., Kennewick, Washington, USA). Most studies used a figure-of-eight shaped coil with a double 70 mm winding or a round coil with a single 90 mm winding. A figure-of-eight shaped coil with a double 50 mm [20] and 25 mm [21] winding, a round coil with a single 120 mm winding [22] and a parabolic coil type [23] were only sporadically used. A few studies did not specify the type of stimulator or coil. Table 3 shows the overview of targeted muscles as well as of stimulators and coils used.

### Participants

Overall, 1978 stroke patients and 377 healthy controls were enrolled. Table 1 summarizes patients characteristics.

#### Patient gender

Five studies (n = 100) did not report data about gender of the included subjects. All remaining studies included mixed patient cohorts. Overall, 1205 males and 674 females were investigated.

#### Time since stroke

time since incident varied considerably among study cohorts (between < 1 day and 16 years at mean). 14 studies (n = 444) tested stroke subjects in the acute phase (within 2 weeks since symptom onset). 20 studies (n = 353) included stroke patients in the subacute phase (2 weeks to 2 months since symptom onset). 59 studies (n = 1182) investigated stroke subjects in the chronic phase (more than 2 months since symptom onset).

#### Stroke etiology

24 studies (n = 498) did not report data about stroke etiology. 19 studies investigated mixed (ischemic and hemorrhagic) patient cohorts. The remaining 49 studies included ischemic stroke subjects only. Overall, 1345 patients with an ischemic stroke and 135 patients with a hemorrhagic stroke were enrolled.

#### Stroke location

24 studies (n = 662) did not report data about stroke location. Most remaining studies included patients with a subcortical stroke as well as patients with a cortical involvement. Overall, 772 patients with a subcortical stroke and 545 patients with a cortical involvement were investigated.

#### Site of lesion

Information about the site of the lesion was absent in 11 studies (n = 352). The remaining studies included 744 right hemispheric and 881 left hemispheric stroke patients.

#### Motor function of the affected hand

Table 2 summarizes mean values of hand motor function tests and their laterality quotients across studies. There was a wide spectrum of motor disability of the affected hand across studies. The laterality quotient varied between 100 (severe hand impairment) and 1 (mild hand impairment).

#### Resting motor threshold

Table 3 summarizes mean values of resting motor threshold and their laterality quotients across studies. Cortical excitability of the ipsilesional and the contralesional hemisphere, as well as the between-hemispheric balance varied strongly across studies.

### Relationships between hand motor impairment/hand motor recovery and rMT

#### Ipsilesional hemisphere

**Fig. 2:**
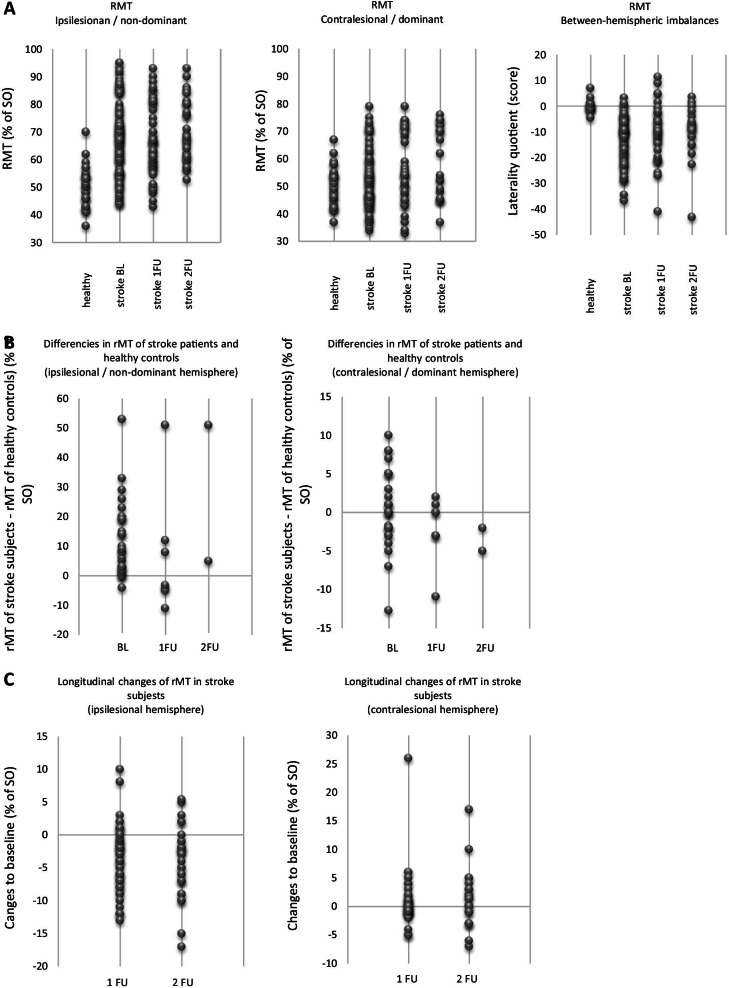
**A** Overall data on resting motor threshold in healthy subjects and stroke patients. Negative values of laterality quotient are associated with a between-hemispheric imbalance towards the contralesional (dominant) hemisphere, positive values with a between-hemispheric imbalance towards the lesioned (non-dominant) hemisphere; **B** Resting motor threshold in stoke patients in comparison to healthy controls (only for studies which included healthy control group). Positive values re associated with a higher, negative values with a lower resting motor threshold in stroke patients (in comparison to healthy controls); **C** Longitudinal changes of rMT in stroke patients. Positive values are associated with an increase, negative values with a decrease of resting motor threshold over time. Notes: BL = baseline; RMT/rMT = resting motor threshold; SO = stimulator output; 1 FU = first follow-up; 2 FU = second follow-up

Figure 2A illustrates overall data on ipsilesional rMT in stroke subjects and non-dominant rMT in healthy controls. Figure 2B demonstrates direct comparison of ipsilesional rMT in stroke patients and non-dominant rMT in healthy controls for studies that included healthy control group. Both illustrations indicate that ipsilesional rMT is increased in most stroke patients. Significant correlations were found between ipsilesional rMT and the amount of hand motor disability at BL (r = 0.558, p < 0.001) and 1 FU (r = 0.359, p = 0.011) in stroke subjects. Furthermore, the amount of increase (in comparison to healthy) correlates with hand motor disability on BL (r = 0.587, p = 0.001) and 1 FU (r = 0.884, p = 0.008). Thus, the higher the hand disability, the stronger the increase of ipsilesional rMT.

Longitudinal data shows a decrease of ipsilesional rMT over time in most of the studies (Fig. 2C). Significant correlations were found between changes of ipsilesional rMT and changes of hand disability from BL to 1 FU (r = 0.326, p = 0.024) and from BL to 2 FU (r = 0.365, p = 0.050). A favorable hand motor recovery was associated with a decrease, an unfavorable recovery with an increase of ipsilesional rMT.

#### Contralesional hemisphere

Figure 2A shows overall data on contralesional rMT in stroke subjects and dominant rMT in healthy controls. Figure 2B demonstrated a direct comparison of contralesional rMT in stroke patients and dominant rMT in healthy controls for studies that included healthy control groups. The illustrations indicate both an increase and a decrease of contralesional rMT in stroke subjects in comparison to healthy subjects. No significant correlations were found between contralesional rMT (or the amount of its changes in comparison to healthy) and the amount of hand motor disability on BL and both FUs.

Longitudinal data demonstrated both an increase and a decrease of contralesional rMT over time (Fig. 2C). No significant correlations were found between changes of contralesional rMT and hand motor recovery.

#### Between-hemispheric imbalance

Most studies show a between-hemisphere imbalance of rMT in favor of the contralesional hemisphere in stroke patients (Fig. 2A). Its amount correlates significantly with hand motor disability at baseline (r = −0.543, p < 0.001). The poorer the motor function of the affected hand, the greater the between-hemispheric imbalance to the disadvantage of the ipsilesional hemisphere. In contrast, mild hand impairment is associated with a slight interhemispheric imbalance towards the ipsilesional hemisphere.

Longitudinal data demonstrates either partial or complete recovery of between-hemispheric balance of rMT over time in most studies (Fig. 2A). However, no significant correlations to hand motor recovery were detected.

#### Relationships between ipsilesional rMT, contralesional rMT and between-hemispheric imbalance of rMT

**Fig. 3:**
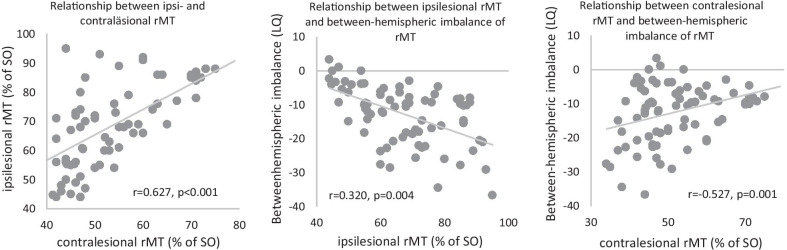
Relationships between ipsilesional rMT, contralesional rMT and between-hemispheric imbalance of rMT at the baseline. Notes: rMT = resting motor threshold; SO = stimulator output

Significant correlations were found between ipsilesional rMT and contralesional rMT at BL (r = 0.627, p < 0.001), 1 FU (r = 0.520, p = 0.001) and 2 FU (r = 0.472, p = 0.031). The higher the ipsilesional rMT, the higher the contralesional rMT (Fig. 3).

Ipsilesional rMT correlated significantly with the laterality quotient of rMT at BL (r = −0.527, p = 0.001) and 1 FU (r = −0.418, p = 0.011). The higher the ipsilesional rMT, the greater the between-hemispheric imbalance to the disadvantage of the ipsilesional hemisphere (Fig. 3).

Contralesional rTMS correlated significantly with the laterality quotient of rMT at baseline (r = 0.320, p = 0.004), 1 FU (r = 0.546, p = 0.001) and 2 FU (p = 0.670, r = 0.001). High contralesional rMT was associated with small between-hemispheric imbalance (Fig. 3).

## Discussion

This systematic review aims to evaluate the neural background of hand motor disability/hand motor recovery in stroke patients, based on resting motor threshold data. In total, 92 studies including 1411 stroke subjects and 331 healthy controls were enrolled and analyzed. The available data demonstrates several relevant relationships between the neurophysiological and the behavioral data. These results may contribute to a better understanding of the neural background of motor recovery after a stroke and support the development of innovative therapies in this cohort.

### Cortical excitability during motor recovery after stroke

Our data shows that severe hand motor impairment in stroke patients is associated with a suppressed cortical excitability within the ipsilesional hemisphere as well as with between-hemispheric imbalance to the disadvantage of the ipsilesional hemisphere. A favorable motor recovery is associated with an increase of ipsilesional cortical excitability and with a reduction of this between-hemispheric imbalance. Completely recovered patients show ipsilesional cortical excitability and between-hemispheric balance comparable to healthy controls. These findings are supported by individual studies reported in our review. Nineteen studies demonstrate within their patients cohort, (1) that low ipsilesional cortical excitability is associated with poor motor function and/or (2) that favorable hand motor recovery is associated with an increase of ipsilesional cortical excitability [20, 21, 23–39]. Similarly, ten trials indicate that large between-hemispheric imbalance to the disadvantage of the ipsilesional hemisphere is associated with severe hand motor impairment, and slight between-hemispheric imbalance in favor of the ipsilesional hemisphere is associated with mild hand impairment [21, 32, 36, 38, 40–44].

With regards to the contralesional hemisphere, our data reveals both higher and lower cortical excitability in stroke patients in comparison to healthy subjects. Nonetheless, the correlation analyses show no significant link to hand motor impairment/hand motor recovery. However, three of the studies (included in our review) found significant relationships in this regard [24, 34, 45]. On the one hand, severely impaired patients in the acute phase (10 days after symptom onset) showed an increase of cortical excitability in both the contra- and the ipsilesional hemisphere, in the course of hand motor recovery [34]. On the other hand, moderately impaired patients in the chronic phase (5 months after the incident) demonstrated a decrease of contralesional cortical excitability over time [45]. Furthermore, chronic stroke patients (> 6 months after the incident) with mild residual hand impairment showed higher contra- and ipsilesional cortical excitability in comparison to severely affected patients [24].

### Cortical excitability versus neuroimaging

**Fig. 4:**
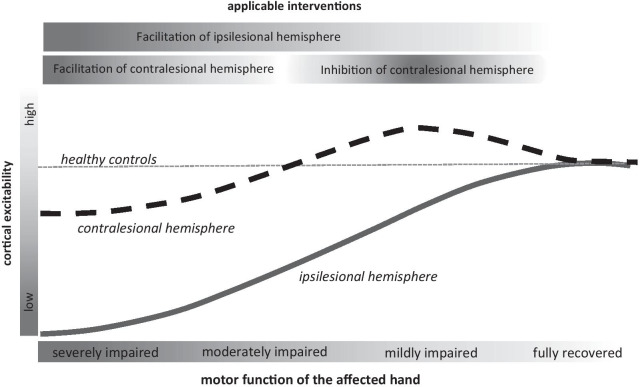
Illustration of (1) changes of the cortical excitability within the ipsilesional and the contralesional hemisphere during motor recovery after a stroke as well as of (2) applicable therapy strategies

Figure 4 illustrates the evolution of cortical excitability in the course of hand motor recovery after stroke, as measured with resting motor threshold data. These observations receive support from a previous systematic review that investigates the neural background of stroke motor recovery with regard to the size and location of hand motor representation as measured by TMS [46].

Our findings differ somewhat from the traditional view of neural processing after stroke on the basis of fMRI and PET data [11–13]. A longitudinal fMRI study demonstrated in severely impaired patients a bilateral increase of task-related neural activation within motor areas during the first two weeks after stroke. The movement-related BOLD activity in mildly impaired stroke patients did not differ from healthy subjects [11]. A cross-sectional study that recruited patients at least three months after stroke revealed similar results. Patients with less favorable hand motor recovery were more likely to recruit several motor-related brain regions over and above those recruited in healthy controls during a hand motor task [12]. In contrast, patients with favorable hand motor recovery showed a brain activation pattern similar to that found in healthy subjects [12]. Motor outcome correlated negatively with task-related activation in various brain regions, such as supplementary motor area, cingulate motor area, premotor cortex, posterior parietal cortex, and cerebellum of both ipsilateral and contralateral hemispheres [12]. In accordance with this data, a PET study demonstrated a significant increase of cerebral blood flow in several brain regions of both the contralateral and ipsilateral hemispheres (primary sensorimotor cortex, cerebellar hemispheres, insular cortex, inferior parietal, and premotor cortices) when stroke survivors moved their affected hand [13]. In contrast, active movement of the non-affected hand was associated with a significant increase of regional cerebral blood flow within the contralateral primary sensorimotor cortex and the ipsilateral cerebellar hemisphere [13]. Taken together fMRI and PET data showed a profound lateralization of neural activation within motor areas of the contralateral hemisphere in healthy subjects moving one hand. Similar brain activation patterns were found in stroke subjects moving a mildly impaired hand. Severe hand motor impairment was associated with increased neural activation within both the contralesional and ipsilesional hemispheres, which deceased over time when motor recovery proceeded. Up to now, it is still not clear if the increased compensatory recruitment of intact brain regions is an effective strategy to overcome motor impairment. A stroke incident activates a cascade of cellular and molecular processes within the peri-lesional tissue and remote brain regions [47]. Initial loss of functional and structural integrity of neural networks is followed by sprouting of axons and dendrites and formation of new synapses. The “rewiring” of neurons is expected to compensate for the stroke-induced loos of brain tissue [48]. However, aging-related decline of neural processing, such as dysfunctional activation spreading [49, 50] or poor network segregation [51, 52] may interfere with an efficient reorganization of the neural network. Elderly people, for example, show less segregated functional networks in comparison to young elderly. Multiple studies indicate the existence of multiple segregated functional networks within the human brain that exhibit correlated activity and are assumed to be functionally connected [53]. Young adults demonstrate quite dense connections within these functional networks and more sparse connections between different networks. In contrast, elderly people show weaker functional connectivity within the same functional network but stronger functional connectivity between regions belonging to different networks [52]. This phenomenon may be the reason for the increase recruitment of contralesional brain regions after stroke. It has been repeatedly demonstrated that a less segregated brain network is associated with worse motor and cognitive performance, independent of age [51, 52]. An important and potentially causal role in this context plays the brain's major inhibitory neurotransmitter, gamma aminobutyric acid (GABA). Present data demonstrates reduced GABA levels in elderly people, which is correlated with both less segregated sensorimotor networks and worse sensorimotor performance in comparison to young adults [51]. The GABAeric system in particular plays a crucial role during the repair phase of stroke [54]. Another cause of extensive network activation in stroke patients may be dysfunctional activation and deactivation of specific brain areas as a result of aging. Young adults show task-related activation (increase of signal) in specific brain regions, and simultaneously deactivation (decrease of signal) in other areas as detected by PET and fMRI [49, 50]. Interestingly, consistent deactivation patterns (within large areas of the lateral parietal cortex, medial parietal, and medial frontal cortex) can be observed across a wide range of tasks and stimulus modalities [50, 55]. A hypothesis suggests that these regions constitute a “default network” which is active when a person is not focused on the outside world, e.g., during remembering, thinking about the future, and mind wandering [50, 56]. Elderly people show in comparison to young adults an increased spread of activation within the “task-positive areas” but a reduced spread deactivation within the “task-negative network” [49]. Such changes are typically explained as upregulation of resources, or alternatively as the reduced suppression of distracting mental processes.

In accordance with our findings, some reviews on this topic question the general validity of the simplified interhemispheric competition model—which posits that suppressing the excitability of the contralesional hemisphere will enhance recovery by reducing interhemispheric inhibition of the stroke hemisphere [57, 58]. An earlier review, for example, analyzed the proposed mechanisms of synaptic and functional reorganization after stroke and suggests a bimodal balance–recovery model that links interhemispheric balancing and functional recovery to the structural reserve (i.e., remaining functional motor output) spared by the lesion [57]. Another review focused on the role of ipsilateral motor pathways during stroke recovery and its implications for non-invasive brain stimulation. Its results emphasize that contralesional M1 suppression may also reduce excitability of ipsilateral descending pathways that may be important for paretic upper limb control for some patients [58].

## Conclusions

This review provides information about the relationship between hand motor function and motor cortex excitability changes within and across both hemispheres during recovery. In particular, the amount of motor cortex excitability of both hemispheres depended on the amount of hand motor function. In comparison to cortical excitability within the ipsilesional hemisphere, which was uniquely suppressed, motor cortex excitability within the contralesional hemisphere was reduced in those with severe hand dysfunction but enhanced in those with a less severe motor disability. Based on these findings, specific rehabilitation approaches may be developed to account for these differential changes in motor cortex excitability for mildly and severely affected stroke subjects. For example, more disabled patients may benefit from therapy strategies, which enhance motor cortex excitability within both hemispheres, e.g., a bilateral hand motor training. In contrast, mildly impaired patients may benefit from strategies that enhance motor cortex excitability within the ipsilesional hemisphere but suppress excitability within the contralesional hemisphere. This may be achieved by constraint induced movement therapy [59]. Also, within the context of non-invasive brain stimulation, the present set of data may be beneficial to develop a specific application of these techniques in dependence of the individual time-point and extent of hand motor recovery. Figure 4 illustrates how inhibitory or facilitatory rehabilitation techniques may be used in a specific fashion depending on the amount of motor impairment of the affected hand during recovery after stroke.

## Strength and limitations

This is the first systematic review on rMT and hand motor function in stroke subjects. Thus, its results may contribute to a better understanding of the neural principles of motor recovery after stroke and support the application of appropriate therapeutical strategies. However, our analysis has limitations related to the reviewed data: i.e., the inconsistency of methods (diverse hand motor assessment scores, different targeted muscles, different types of stimulators and coils), subjects (different stroke states, etiologies, locations), and study designs (observational versus interventional studies, different follow-up timings). This may hamper the interpretation of the results.

## Data Availability

The datasets generated during and/or analyzed during the current study are available from the corresponding author on reasonable request.

## Abbreviations

BOLD: Blood-oxygen-level-dependent
BL: Baseline
(f)MRI: (Functional) magnetic resonance imaging
GABA: Gamma aminobutyric acid
MEP: Motor evoked potential
Mm: Millimeter
M1: Primary motor cortex
PET: Positron emission tomography
RMT/rMT: Resting motor threshold
(r)TMS: (Repetitive) transcranial magnetic stimulation
µV: Microvolt
1 FU: First follow-up
2 FU: Second follow-up
